# New Insights from Two Historic *Boletellus*-Type Specimens in China Based on Next-Generation Sequencing

**DOI:** 10.3390/jof12030223

**Published:** 2026-03-19

**Authors:** Bing-Qian Yang, Hong-Kang Shen, Yang Wang, Xin Zhang, Fa-Ming Long, Yi-Lin He, Yan-Chun Li, Gang Wu, Jing Zhou

**Affiliations:** 1 School of Pharmaceutical Science and Yunnan Key Laboratory of Pharmacology for Natural Products, Department of Pharmaceutical Sciences, Kunming Medical University, Kunming 650500, China; 13769853349@163.com (B.-Q.Y.); 14769214805@163.com (F.-M.L.); 2 State Key Laboratory of Phytochemistry and Natural Medicines, Kunming Institute of Botany, Chinese Academy of Sciences, Kunming 650201, China; shenhongkang@mail.kib.ac.cn (H.-K.S.); lesireyang@163.com (Y.W.); heyilin@mail.kib.ac.cn (Y.-L.H.); liyanch@mail.kib.ac.cn (Y.-C.L.); 3 Yunnan Key Laboratory for Fungal Diversity and Green Development, Kunming 650201, China; 4 College of Life Sciences, University of Chinese Academy of Sciences, Beijing 100049, China; 5 College of Horticulture and Landscape, Yunnan Forestry Technological College, Kunming 650224, China; zhangxin8191@163.com; 6 College of Biological and Food Engineering, Southwest Forestry University, Kunming 650233, China

**Keywords:** *Boletellus*, genome-skimming, molecular phylogeny, taxonomic revision

## Abstract

*Boletellus* is a morphologically distinctive genus within the family Boletaceae, characterized by basidiospores with longitudinally striate ornamentation. Although the species diversity of this genus in China has been well documented in recent years, several historically described species published by early Chinese mycologists have been largely overlooked. To clarify the taxonomic identities of these historic *Boletellus* species from China, this study applied a genome-skimming approach to perform next-generation sequencing (NGS) on the historical type specimens of *B. serpentipileus* and *B. vulgaris.* The integration of NGS data with Sanger sequencing and morphological re-examination enabled a comprehensive taxonomic reassessment, which revealed that *B. serpentipileus* and *B. vulgaris* are not members of *Boletellus*, but belong to *Leccinum* and *Austroboletus*, respectively. Accordingly, the new combination *Leccinum serpentipileum* is proposed, and *B. vulgaris* is further synonymized with *A. fusisporus.* These findings resolve long-standing taxonomic uncertainties and contribute to a more accurate understanding of bolete diversity in China.

## 1. Introduction

The genus *Boletellus* was originally established by Murrill [1], based on collections from the southeastern USA, to accommodate *Boletus ananas* [2]. The diagnostic features of the genus include the pileus covered with erect, conical, or appressed fibrillose scales; context typically yellow and often bruising blue or sometimes unchanged; and basidiospores bearing striate ornamentation [3,4]. According to the recent research by Xue et al. (2023) [5], there were 17 species of *Boletellus* recorded in China. However, due to limited study of type specimens of some early described species, the true species diversity within this group remains to be clarified. For example, despite being described decades ago, the two *Boletellus* species, namely *B. serpentipileus* [6] and *B. vulgaris* [7], have not been revisited, leaving their true taxonomic positions unresolved.

Genome skimming is a low-coverage whole-genome sequencing strategy based on next-generation sequencing (NGS) technology, enabling efficient retrieval of phylogenetic marker genes and genomic fragment information [8]. Unlike traditional genome sequencing methods, which require complex sample preparation and involve high-cost procedures, genome skimming can obtain genomic data more rapidly and at a lower cost [9]. This approach holds significant potential to become a mainstream technology in genomic research and has been increasingly applied in recent years to fungal systematics and taxonomic identification studies [10,11]. This potential makes it possible to revisit early described *Boletellus* species, which were originally defined solely by morphological data.

By integrating NGS data with conventional morphological and molecular evidence, this study aims to (1) clarify the phylogenetic placements of *B. serpentipileus* and *B. vulgaris*; (2) evaluate the consistency between their morphological characteristics and molecular phylogenetic relationships; and (3) to propose the potential taxonomic revisions.

## 2. Materials and Methods

### 2.1. Morphological Characterization

Voucher specimens in this study were deposited in the Herbarium of Cryptogams at the Kunming Institute of Botany, Chinese Academy of Sciences (KUN-HKAS), and the Fungarium of the Guangdong Institute of Microbiology (GDGM). Macroscopic descriptions were based on field notes and digital images of basidiomata. Microstructural features were examined by light microscopy, whereas basidiospore ornamentation was observed under a scanning electron microscope (SEM) ZEISS Sigma 300 [12]. Tissue sections were prepared freehand, rehydrated in 5% KOH solution (Guangdong Guanghua Sci-Tech Co., Ltd., Guangzhou, Guangdong, China), and stained with 1% Congo red (Tianjin Guangfu Technology Development Co., Ltd., Tianjin, China) when necessary. Photomicrographs were captured using a ZEISS Axio Scope A1 microscope (ZEISS Technology (Suzhou) Co., Ltd., Suzhou, China) equipped with a Motic MG T45 digital camera system. Basidiospore measurements are expressed as “n/m/p”, indicating that n basidiospores were measured from m basidiomata of p collections. Spore dimensions (length/width) are presented as “(a–)b–c(–d)”, where the range b–c contains 90% of the measured values, with the extreme values a (minimum) and d (maximum) shown in parentheses. The length/width ratio of basidiospores is denoted by “Q”, while “**Q**” (in bold) represents the arithmetic mean ± standard deviation of the basidiospore length/width ratios.

### 2.2. DNA Extraction and Sequencing Methods

This study employed both Sanger sequencing and next-generation sequencing techniques. Sanger sequencing was preferentially applied to all specimens; however, for those yielding poor sequencing quality, NGS was subsequently employed. In the Sanger sequencing process, total genomic DNA was extracted from dried samples using the Ezup Column Fungal Genomic DNA Purification Kit (Sangon Biotech Co., Ltd., Shanghai, China), from materials dried according to the manufacturer’s instructions. The nuclear rDNA region, comprising the internal transcribed spacers 1 and 2 with the 5.8S rDNA (ITS) and the nuclear ribosomal large subunit (nrLSU), were amplified using the universal primer pairs ITS1-F (or ITS5) + ITS4 [13,14] and LROR + LR5 [15], respectively. For amplification of partial sequences of the RNA polymerase II largest subunit gene (*rpb1*), the RNA polymerase II second largest subunit gene (*rpb2*), the second-largest subunit (*rpb2*), and the translation elongation factor 1-α gene (*tef1-α*), the specific primers RPB1-B-F + RPB1-B-R (or RPB2-B-F2 + RPB2-B-R) and EF1-B-F2 + EF1-B-R, designed by Wu et al. (2014) [16], were employed. PCR amplification was performed using the following reaction system (25 µL total volume): 12.5 µL of 2× Hieff^®^ PCR Master Mix (Yeasen Biotechnology (Shanghai) Co., Ltd., Shanghai, China), 9.5 µL of ddH_2_O (Sangon Biotech (Shanghai) Co., Ltd., Shanghai, China), 1 µL each of forward and reverse primers (100 µM), and 1 µL of total DNA solution. The reaction conditions were as follows: initial denaturation at 94 °C for 4 min, followed by 35 cycles of denaturation at 94 °C for 60 s, annealing at 48 °C, 52 °C, or 53 °C for 60 s, and extension at 72 °C for 80 s, with a final extension at 72 °C for 8 min. The PCR products were resolved by electrophoresis on 1% agarose gels in 1× TAE buffer. Amplification results were visualized and documented using a gel imaging system. Positive reactions containing the target band were then sequenced on an ABI-3730-XL DNA Analyzer (Applied Biosystems, Foster City, CA, USA).

Next-generation sequencing was applied to historical type specimens (more than 20 years old) of *B. serpentipileus* (KUN-HKAS31116) and *B. vulgaris* (GDGM4454) and conducted on the MGI DNBSEQ-T7 platform, generating about 5 Gb of sequencing data per sample. For genomic data analysis, raw reads were quality-controlled using FASTP v1.0.164 (Shenzhen Institutes of Advanced Technology, Chinese Academy of Sciences, Shenzhen, China) [17] and assembled with SPAdes v4.2.065 (Saint Petersburg State University, St. Petersburg, Russia) [18] under default parameters. Detailed procedures, parameter settings, and result interpretations for quality control and assembly, respectively, are available at the following links: https://github.com/OpenGene/fastp and https://github.com/ablab/spades (accessed on 1 March 2025). Subsequently, library comparisons were performed, and the target fragments—ITS, nrLSU, *tef1-α*, *rpb1*, and *rpb2*—were extracted from the assembled genome contigs [19]. The obtained sequences were double-checked using NCBI’s BLAST (available online: https://blast.ncbi.nlm.nih.gov/Blast.cgi, accessed on 20 March 2025) to assess their reliability. Through this approach, a total of 7 sequences were obtained for the aforementioned two species. The specific operational procedures for sequence extraction are as follows:

# Build a comparison database

makeblastdb -in ../contigs.fasta -dbtype nucl -parse_seqids -out index

# Target fragment extraction analysis

time blastn

-db index

-query 1.fas

-outfmt ‘6 delim= @ sseq’

-num_threads 18 -out 2.fas

# 1.fas: sequence file for comparison

# 2.fas: output file for target sequences

### 2.3. Sequence Alignment and Matrix Assembly

For the assembled genomic data, we used BUSCO v5.5.0 (University of Geneva, Geneva, Switzerland) [20] to assess the assembly quality of the obtained contigs.fasta. Since the specimens belong to the order Boletales, the “boletales_odb10” lineage-specific dataset was utilized for this assessment. In this study, due to the poor sequencing quality of both the type specimen *B. serpentipileus* (KUN-HKAS31116) and the type specimen *B. vulgaris* (GDGM4454), the proportion of complete single-copy orthologous genes was relatively low, which was insufficient to support the construction of a phylogenomic tree. Therefore, we instead employed a method involving the selection of gene sequence fragments for tree reconstruction.

For the sequences obtained in this study, we conducted preliminary analysis using the BLASTn program of the GenBank database. Results with sequence coverage exceeding 90% and similarity above 96% were selected, with one to three representative sequences chosen per species. These downloaded sequences were merged with those we newly generated. For the genus *Leccinum*, phylogenetic analysis was conducted using four gene matrices—nrLSU, *tef1-α*, *rpb1*, and *rpb2*. ITS sequences were excluded due to their excessive variation among *Leccinum* species [21]. For the genus *Austroboletus*, five matrices were generated: ITS, nrLSU, *tef1-α*, *rpb1*, and *rpb2*. The following steps on phylogenetic analysis were performed using PhyloSuite v1.2.3 (Institute of Hydrobiology, Chinese Academy of Sciences, Wuhan, China) [22]: sequence alignment was performed with MAFFT v7.313 (Immunology Frontier Research Center, Osaka University, Osaka, Japan) using the simplest FFT-NS-1 strategy [23]; aligned sequences were trimmed using the “manual trim” mode of trimAl v1.2 (Centre for Genomic Regulation, Barcelona, Spain) (command line parameters: -cons 25.0 -gt 0.9 -st 0.0 -w 1.0) [24]; and the “Concatenate Sequence” tool was used to merge multiple sequences in a specified order to generate a new, complete concatenated sequence.

### 2.4. Molecular Phylogenetic Analyses

For the combined dataset, molecular phylogenetic analyses were conducted using Maximum Likelihood (ML) and Bayesian Inference (BI) methods. To ensure the biological rationality of the combined analysis, phylogenetic trees were first constructed separately for each gene. Topological congruence was assessed based on the criterion proposed by Nuhn et al. (2013) [25], which considers clades with conflicting topologies supported by bootstrap values >70% as significantly incongruent. No significant topological conflicts were detected among the individual gene trees; therefore, the sequences were concatenated for subsequent joint analyses. Maximum Likelihood analysis was performed using RAxML v8.2.10 (Heidelberg Institute for Theoretical Studies, Heidelberg, Germany) [26] called via the graphical interface raxmlGUI 2.0.0-beta.3 (University of Gothenburg, Gothenburg, Sweden) [27]. The substitution model was set to GTRGAMMAI, with all other parameters kept at their default settings. Bootstrap support values were obtained from 1000 non-parametric bootstrap replicates. All analyses were run on a Windows system utilizing 4 CPU threads. Bayesian Inference Analysis was carried out using PhyloSuite v1.2.3 according to the following workflow: Prior to analysis, PartitionFinder 2 (Australian National University, Canberra, Australia; Macquarie University, Sydney, Australia) [28] was used to select optimal partitioning schemes and substitution models under the corrected Akaike Information Criterion (AICc). The search was conducted using a greedy algorithm with the candidate model set comprising 24 nucleotide substitution models (ranging from JC to GTR + I + G). The model selection results indicated the following best-fit models: for the genus *Leccinum* matrix, GTR + I + G for nrLSU, SYM + G for *tef1-α* and *rpb1*, and K80 + G for *rpb2*; for the genus *Austroboletus* matrix, GTR + I + G for ITS and nrLSU, SYM + G for *tef1-α* and *rpb2*, and HKY + I + G for *rpb1*. Bayesian Inference was subsequently executed in MrBayes v3.2.6 (Swedish Museum of Natural History, Stockholm, Sweden; Florida State University, Tallahassee, FL, USA) [29]. The evolutionary model for each partition was specified via the nst and rate parameters to correspond with the optimal models previously identified by PartitionFinder 2. Two independent runs were performed simultaneously, each consisting of one cold chain and three heated chains (four chains in total). The analysis was run for 1.0 × 10^6^ generations, sampling tree topologies and model parameters every 1000 generations. MrBayes utilizes Markov Chain Monte Carlo (MCMC) for Bayesian Phylogenetic Inference. The ideal state of the MCMC run is to reach the target posterior probability distribution. During execution, the “Progress” window displays the Average Standard Deviation of Split Frequencies (ASDSF) in real time. An ASDSF value below 0.01 was considered indicative of convergence. If the run terminated without achieving convergence, the analysis was extended until convergence was reached, after which the final result file (con.tre) was obtained. For this study, both the *Leccinum* dataset and the *Austroboletus* dataset were ultimately run for 2.0 × 10^6^ generations.

## 3. Results

### 3.1. Molecular Phylogeny

**Figure 1:**
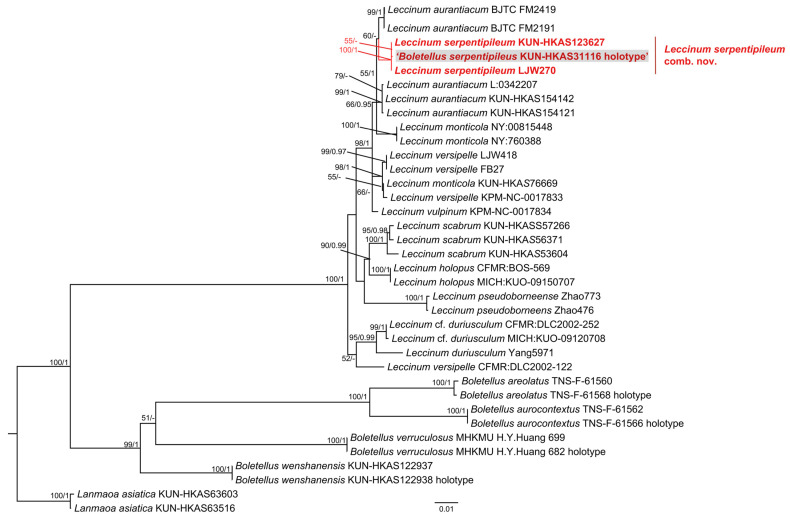
Maximum Likelihood phylogenetic tree inferred from a multi-gene dataset (nrLSU, *tef1-α*, *rpb1*, and *rpb2*), showing the phylogenetic placement of *B. serpentipileus*. The tree was rooted with *Lanmaoa asiatica* (HKAS63516) and *Lanmaoa asiatica* (HKAS63603). Node support values are indicated above or adjacent to the corresponding branches, showing Maximum Likelihood Bootstrap support ≥50% (ML-BS ≥ 50%) and Bayesian Posterior Probabilities ≥ 0.90 (BI-PP ≥ 0.90). The type specimen of *B. serpentipileus* is highlighted within a gray box.

**Figure 2:**
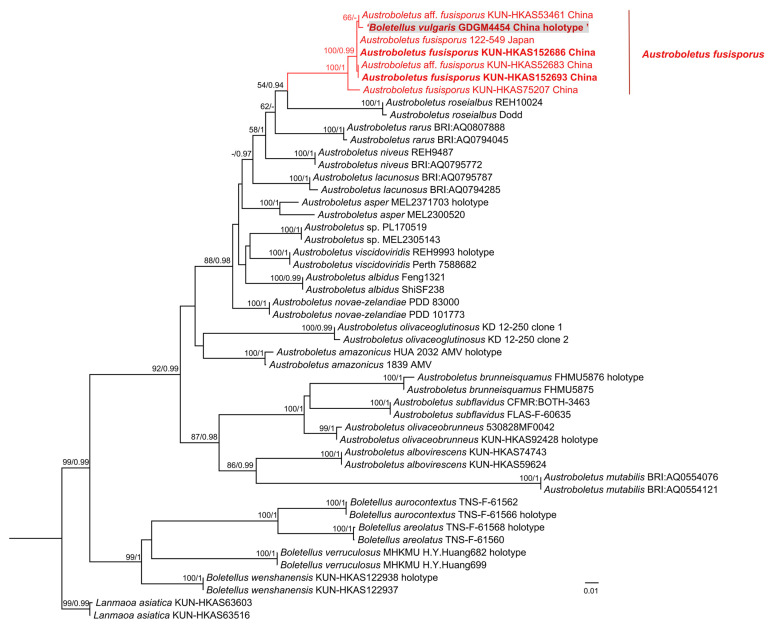
Maximum Likelihood phylogenetic tree inferred from a multi-gene dataset (ITS, nrLSU, *tef1-α*, *rpb1*, and *rpb2*), showing the phylogenetic placement of *B. vulgaris*. The tree was rooted with *Lanmaoa asiatica* (KUN-HKAS63516) and *Lanmaoa asiatica* (KUN-HKAS63603). Node support values are indicated above or adjacent to the corresponding branches, showing ML bootstrap support ≥50% (ML-BS ≥ 50%) and Bayesian Posterior Probabilities ≥ 0.90 (BI-PP ≥ 0.90). The type specimen of *B. vulgaris* is highlighted within a gray box.

In this study, a total of 29 sequences were newly generated, comprising five ITS, seven nrLSU, six *tef1-α*, two *rpb1*, and five *rpb2* sequences. Specimens KUN-HKAS152686, KUN-HKAS152693, and KUN-HKAS154141 were sequenced using the Sanger method. Specimens KUN-HKAS31116, GDGM4454, and KUN-HKAS154142 were sequenced using NGS. For specimen KUN-HKAS123627, the ITS and nrLSU sequences were obtained via Sanger sequencing, while the *tef1-α, rpb1*, and *rpb2* sequences were obtained via NGS. All of the sequences have been submitted to GenBank (Table 1). For the genus *Leccinum*, a concatenated phylogenetic analysis was performed based on the nrLSU, *tef1-α*, *rpb1*, and *rpb2*; the combined dataset comprised 17 species and 2535 nucleotide sites. For the genus *Austroboletus*, a concatenated phylogenetic analysis was conducted using ITS, nrLSU, *tef1-α*, *rpb1*, and *rpb2*; the combined dataset comprised 22 phylogenetic species and 2616 nucleotide sites. The trees reconstructed using Maximum Likelihood (ML) and Bayesian Inference (BI) methods exhibited similar topologies. Therefore, only the ML trees were presented (Figure 1 and Figure 2).

In the *Leccinum* analysis, we performed ITS sequence alignment for a specimen (KUN-HKAS154142) collected from France. The alignment showed 100% coverage and greater than 99% similarity to that of an authentic *L. aurantiacum* specimen (Champ-41) from France, which is the type locality for this species. Therefore, this specimen (KUN-HKAS154142) was identified as *L. aurantiacum*, and its DNA sequences of other genes were included in the multi-gene (nrLSU + *tef1-α* + *rpb1* + *rpb2*) phylogenetic analysis. The results revealed that the clade containing the type specimen of *B. serpentipileus* (KUN-HKAS31116) was clearly positioned outside the genus *Boletellus*. Instead, it was clustered within *Leccinum* and formed a monophyletic group distinct from *L. aurantiacum* (KUN-HKAS154142) (Figure 1). In the *Austroboletus* analysis, phylogenetic analysis using a multi-gene dataset (ITS + nrLSU + *tef1-α* + *rpb1* + *rpb2*) placed the type specimen of *B. vulgaris* (GDGM4454) outside the genus *Boletellus* and, specifically, within a *Austroboletus* clade alongside *A. fusisporus*. Consequently, based on the molecular phylogenetic results and supported by the morphological evidence (as detailed below), we proposed to transfer *B. serpentipileus* to the genus *Leccinum* as *L. serpentipileum*, and to treat *B. vulgaris* as a synonym of *A. fusisporus.*

### 3.2. Morphological Study of Type Specimens

*Boletellus serpentipileus* was originally published by Zang & Yuan (1999) from Yunnan Province in China, with the original description stating: “Pileus 7~9 cm latus, convexus demum planoconvexus, siccus, rugulosus, reticulato–venosus, brunneo–flavus, serpentiformis. Contextus 1~1.6 cm crassus, albus, immutabilis. Hymenium aureum. Tubuli 0.5~1 cm longi, flavi, adnexi vel sinuato–adnexi. Pori angulares vel irregulares. 14~18 per cm. Stipe 7~9 cm longus. 1.5~2.2 cm crassus, clavatus, reticulatus, basim versus bulbosus, brunneo–flavus. Mycelio albido. Basidiosporae 21~23 × 6~6.5 μm angusto–ellipsoideae, disstincte longitudinalis rugiformis. Basidia 25~30 × 8~10 μm, clavata. 4–sporigera. Tramae tubi paralleloneurae [6].”

**Figure 3:**
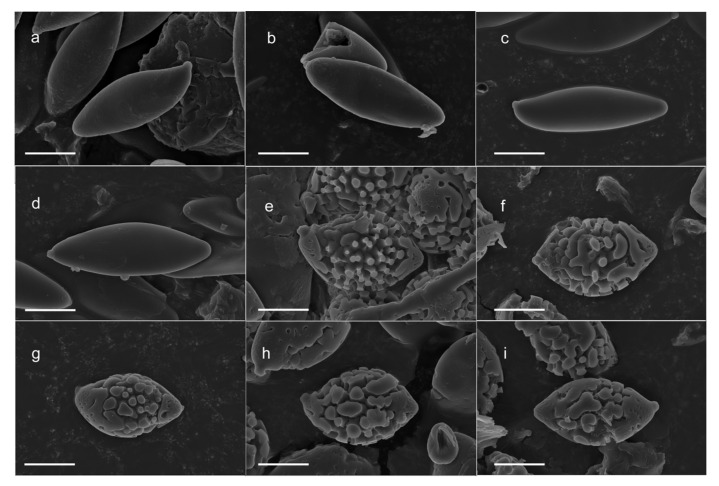
Basidiospores under SEM from voucher specimens of *L. serpentipileum* and *A. fusisporus*. (**a**–**d**) *L. serpentipileum*: ((**a**,**b**) KUN-HKAS31116, holotype; (**c**,**d**) KUM-HKAS123627). (**e**–**i**) *A. fusisporus*: ((**e**,**f**) GDGM4454, holotype of *B. vulgaris*; (**g**) KUN-HKAS152686; (**h**,**i**) KUN-HKAS152693). Scale bars = 5 μm.

**Figure 4:**
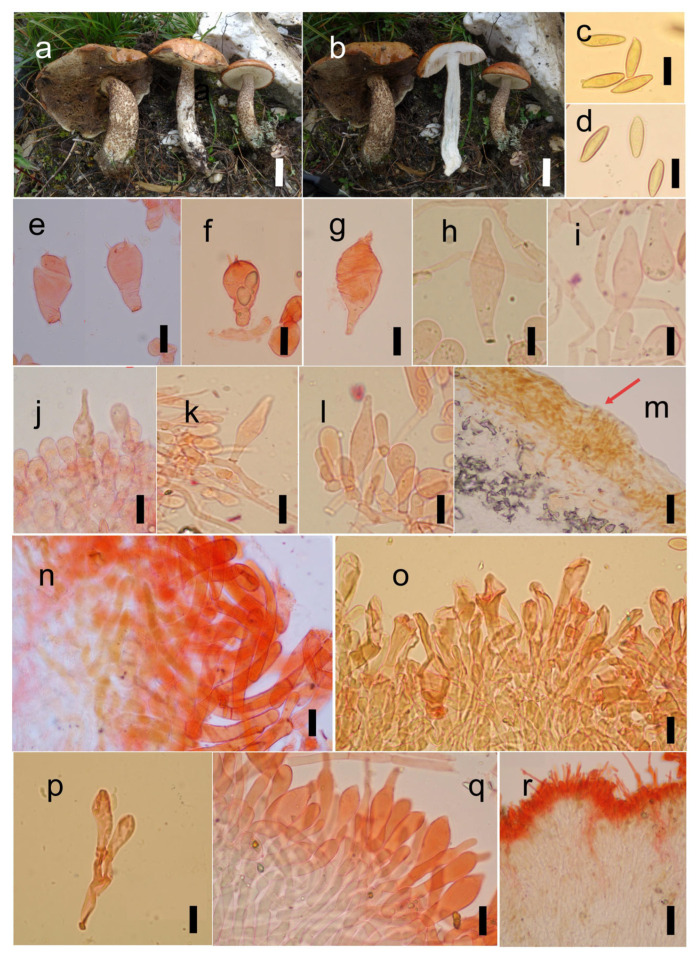
The microscopic characteristics of *L. serpentipileum*: ((**a**,**b**,**d**–**n**,**q**,**r**) KUN-HKAS 123627; (**c**,**o**,**p**) KUN-HKAS31116, holotype). (**a**,**b**) basidiomata, (**c**,**d**) basidiospores, (**e**,**f**) basidia, (**g**–**i**) pleurocystidia, (**j**–**l**) cheilocystidia, (**m**,**n**) pileipellis, (**p**) caulocystidia, and (**o**,**q**,**r**) stipitipellis. Scale bars: (**a**,**b**) = 3 cm; (**c**–**l**,**n**–**q**) = 10 µm; and (**m**,**r**) = 40 µm. The red arrow in subpanel m indicates the gelatinous substance.

Based on the re-examination of the holotype specimen of *B. serpentipileus*, which is in suboptimal condition, the identifiable morphological characteristics are as follows: Basidiomata medium-sized to large. Pileus yellowish brown when dry, stipe surface with squamulose ornamentation, central stipe. Basidiospores (Figure 3a–d and Figure 4c) 16–19 × 4–6 µm, smooth, elongated ellipsoid, with slightly thickened walls (approx. 0.5 µm thick). Basidia 13.5–30 × 8–10 μm, clavate, sterigmata up to 3 μm long, pale yellow in 5% KOH. Cheilo- and pleurocystidia not observed. Hymenophoral trama boletoid. Pileipellis an intricate ixotrichoderm. Stipitipellis hymeniform, approximately 60–90 μm thick; caulocystidia 24–45 × 6–13 μm, broadly fusoid–ventricose, thin-walled. Clamp connections absent in all tissues.

*Boletellus vulgaris* was originally published by Bi et al. (1982) from Guangdong Province in China, with the original description stating: “Pileus 1.6 cm latus, glutinosus, fulvus, margine sordide flavus, dein plano-convexus, villoso-tomentosus, margine porrectus, integris. Contexto albo, immutabili, 3 mm crasso, sapor nullus. Stipes centralis, flavobadius, 5 × 0.3 cm, leviter sinuosus, cylindraceus, solidus, sequalis vel ad basin subincreasatus, tomentosus, cuticula facile delapsus. Tubuli punicei, immutabiles, 3 mm longi, ad stipitem appendiculati, poris angularis, 1 mm diam. Sporae fusiformes, sub microscopio pallide rubropurpureae, verrucosae, 12−17 × 6.6−9 μm. Solitarius ad terram in silva [7].”

**Figure 5:**
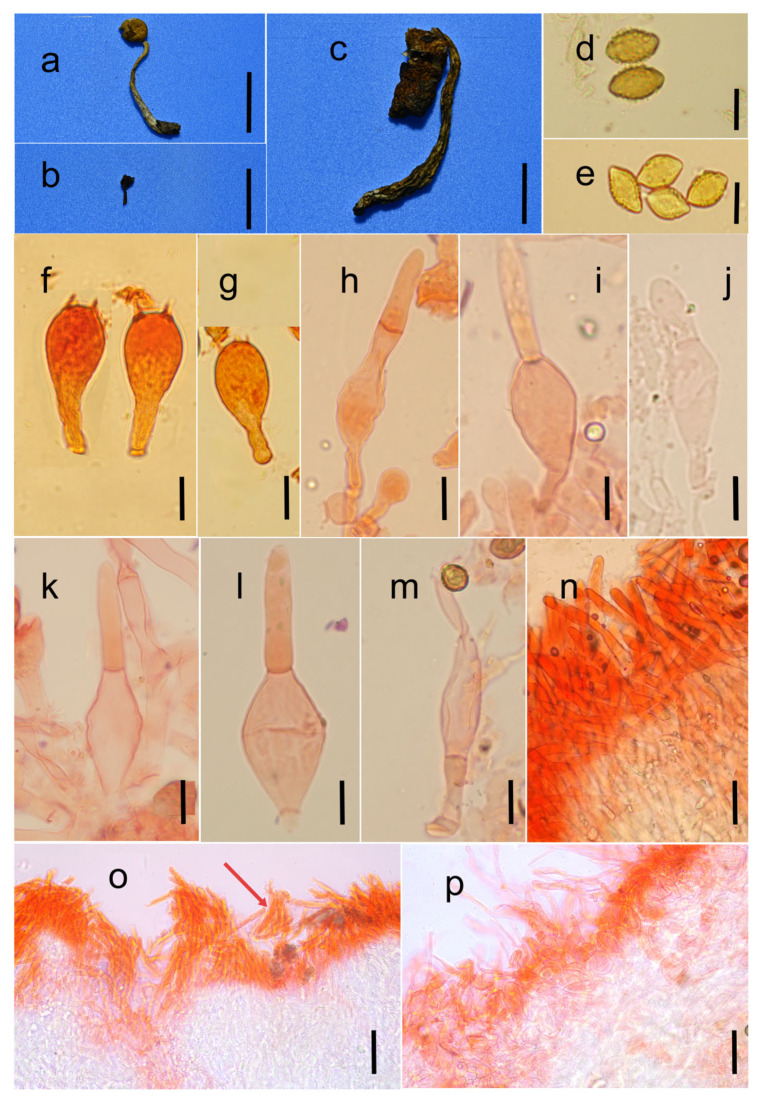
The microscopic characteristics of *A. fusisporus*: ((**a**,**n**–**p**) KUN-HKAS152686; (**b**,**d**) GDGM4454 holotype of *B. vulgaris*; (**c**,**e**–**m**) KUN-HKAS152693). (**a**–**c**) basidiomata, (**d**,**e**) basidiospores, (**f**,**g**) basidia, (**h**–**j**) pleurocystidia, (**k**–**m**) cheilocystidia, (**n**,**o**) pileipellis, and (**p**) stipitipellis. Scale bars: (**a**–**c**) = 2 cm; (**d**–**n**) = 10 um; and (**o**,**p**) = 40 um. The red arrow in subpanel o indicates the gelatinous substance.

Based on the re-examination of the holotype specimen of *B. vulgaris*, which is in suboptimal condition, the identifiable morphological characteristics are as follows: Basidioma small. Pileus fulvous with a central stipe. Basidiospores (Figure 4e–f and Figure 5d) 13–15(–16) × 8–10 μm, verrucose, fusiform, and inequilateral in lateral view with a slightly depressed suprahilar area; smooth or exhibiting shallow pits at the apex and base, appearing brownish yellow in 5% KOH. Basidia 19.5–33 × 8–16.5 μm, clavate, with sterigmata up to 3.5 μm long and pale yellowish in 5% KOH. Cystidia, pileipellis, and stipitipellis not observed.

### 3.3. Taxonomy

*Leccinum serpentipileum* (M. Zang & M.S. Yuan) Bing-Qian Yang & G. Wu, **comb. nov.** (Figure 3a–d and Figure 4).

**Basionym:***Boletellus serpentipileus* M. Zang & M.S. Yuan, Acta bot. Yunn. 21(1): 39 (1999).

**MycoBank:** MB 862341.

**Type:** CHINA, Sichuan Province, Ganzi Tibetan Autonomous Prefecture, Jiulong County, Jichou Mountain, 10 September 1996, Ming-Sheng Yuan 2662 (KUN-HKAS 31116).

**Diagnosis:** Medium to large basidiomata; a pileus surface often orange–yellow and covered with a glutinous tomentum; context white, not discoloring or turning slightly reddish when injured; basidiospores smooth and ellipsoid; and pileipellis an intricate ixotrichoderm.

**Description:** *Basidiomata* medium to large. *Pileus* 6–14 cm in diam., hemispherical; margin slightly incurved, orange–yellow to yellowish brown, covered with yellowish-brown tomentum, viscid when moist; context white, 1–2 cm thick, not discoloring or turning slightly reddish when injured. *Hymenophore* sinuate to adnate, surface initially pale yellow to white, becoming brown at maturity, staining brown when bruised, tubes about 1.5–2 cm long, white to pale yellow when young, becoming yellowish brown at maturity, turning slightly reddish when bruised; pores angular to irregular, 0.5–1 mm wide. *Stipe* central, 7–13 cm long, 1.5–3 cm in diam., solid, often curved, subequal, yellowish to brownish or pallid in the upper part, white to pale yellow in the middle part, gradually whitish towards the base, entirely covered with brown verruculae on the surface; context white, becoming slightly bluish-black when injured; basal mycelium whitish.

*Basidiospores* [42/2/4] (14–)15–18(–19) × 4–5(–6) um [Q = (2.80–)3–4.25(–4.75), **Q** = 3.41 ± 0.40], angusto-ellipsoideae, inequilateral in lateral view with a slight suprahilar depression, elongate ellipsoid in ventral view, yellowish to brownish in 5% KOH, smooth, slightly thick-walled (ca. 0.5 μm thick). *Basidia* 22–34 × 8–18 μm, clavate, four-spored, sterigmata up to 4 µm long, hyaline in 5% KOH. *Hymenophoral trama* boletoid. *Pleurocystidia* 19–44 × 6–17 μm, broadly fusoid–ventricose to subfusiform, with a rounded rostrum at the apex, hyaline in 5% KOH, thin-walled. *Cheilocystidia* 29–59 × 5–16 μm, fusoid–ventricose, subfusiform, or narrowly lageniform, occasionally with an elongated rostrum, slenderer than pleurocystidia, concolorous with pleurocystidia, thin-walled. *Pileipellis* an intricate ixotrichoderm, embedded in a gelatinous matrix, approximately 150 μm thick, composed of interwoven, smooth, thin-walled hyphae 5–10 μm wide; terminal elements 23–64 × 6–15 μm, subcylindrical, light yellowish to brownish. *Stipitipellis* 70–120 μm thick, hymeniform; caulocystidia 27–70(–80) × 4–16 μm, broadly fusoid–ventricose, with a bluntly acute apex or bearing a long beak, thin-walled. *Stipital trama* composed of longitudinally arranged hyphae 5–10 μm diam. *Clamp connections* absent in all tissues of the basidioma.

**Habitat and distribution:** Solitary or scattered on the ground in subalpine coniferous forests; currently known from northwestern Yunnan and western Sichuan, China.

**Additional specimens examined:** CHINA, Yunnan Province, Diqing City, Shangri-La County, alt. ca. 3300 m, 1 September 2017, Gang Wu 2474 (KUN-HKAS 123627); Yunnan Province, Shangri-La County, near the Timber Inspection Station of Daxue Mountain, 8 September 2014, Jian-Wei Liu 270 (KUN-HKAS91078).

*Austroboletus fusisporus* (Kawam. ex Imazeki & Hongo) Wolfe, Biblthca Mycol. 69: 96 (1980) [1979] (Figure 3e–i and Figure 5).

**Basionym:***Porphyrellus fusisporus* Kawam. ex Imazeki & Hongo, Acta phytotax. geobot., Kyoto 18(4): 110 (1960).

**Synonym:***Boletellus vulgaris* C.S. Bi, in Bi, Loh & Zheng, Acta bot. Yunn. 4(1): 57 (1982).

**Diagnosis:** Very small to minute basidiomata; pink hymenophore; stipe covered with distinct pale brown reticula; amygdaliform to fusiform basidiospores ornamented with large, regular to irregular subcylindrical structures; and an ixotrichoderm pileipellis.

**Description:***Basidiomata* small. *Pileus* 1.6–3 cm in diam., glutinous, fulvous (tawny), paler towards margin; becoming plano-convex, villose–tomentose; margin extended, forming a membranous veil that embraces the stipe at the young stage, then fragmented and appendiculate at the margin when aged; context white, 0.3–0.5 cm thick, not discoloring when injured. *Hymenophore* adnate, surface pale pinkish; tubes up to 0.3–0.6 cm long, pink, not discoloring when bruised; pores angular, 1 mm in diam. *Stipe* central, 3–5 cm long, 0.3–0.6 cm in diam., solid, slightly sinuous, cylindrical, equal or slightly thickened at base, yellowish-brown, covered with pale brown reticula on the surface; basal mycelium white.

*Basidiospores* [60/3/3] (10.5–)12–15(–17) × (6–)7–10 um [Q = (1.3–)1.5–1.75(–2), Q = 1.6 ± 0.15], amygdaliform to fusiform, inequilateral in lateral view with a slight suprahilar depression; amygdaliform in ventral view, yellowish to brownish yellow in 5% KOH, ornamented with large, regular to irregular, subcylindrical warts (1–1.5 μm high, 0.5–2 μm wide) on the surface, smooth or with shallow pits at the apex and base. *Basidia* 15–59 × 7–29 μm, clavate, four-spored, sometimes two- or three-spored, sterigmata up to 3.5 μm long, pale yellow in 5% KOH; *Hymenophoral trama* boletoid. *Cheilo-* and *pleurocystidia* consisting of two cells: the upper cells cylindrical to digitate, measuring 17–40 × 3–6.5 μm; the lower cells clavate to broadly clavate, measuring 25–39 × 8–17 μm. These cells contain pale yellowish brown to pale brown pigments, appearing pale brownish yellow to yellowish brown in KOH, and are thin-walled. *Pileipellis* an intricate ixotrichoderm, about 70–165 μm thick, composed of interwoven, smooth, thin-walled hyphae measuring 4–8 μm in diameter; terminal cells 19–78 × 3.5–8 μm, subcylindrical to subclavate, hyphae transparent yellow in 5% KOH. *Caulocystidia* scarce, resembling hymenial cystidia, with the upper cells measuring 12–18 × 3–5 μm and the lower cells 15–30 × 8–12 μm. *Stipitipellis* a hyphoepithelium: the outer layer consists of filamentous hyphae measuring 2–11 μm in width, with clavate to subcylindrical terminal cells sized 15–64 × 5–10 μm, appearing pale yellowish to brownish yellow in KOH. *Clamp connections* absent in all tissues of the basidioma.

**Habitat and distribution:** Scattered on soil in tropical forests dominated by plants of the family Fagaceae; currently known in Japan [45], South Korea [46], and China [4,30].

**Specimens examined:** CHINA, Guangdong Province, Zhaoqing City, near Qingyun Temple on Dinghu Mountain, 19 August 1980, Zhi-Shu Bi 454 (GDGM 4454, holotype of *B. vulgaris*); Hunan Province, Changsha City, Lishanchong, 4 September 2023, Wu-Ping Luo, voucher no. SF1442787849763 (KUN-HKAS 152686); Yunnan Province, Lincang City, 14 July 2024, collected by an anonymous enthusiast, voucher no. SF1454082419825 (KUN-HKAS 152693).

## 4. Discussion

In this study, we re-examined two early described species of *Boletellus*, *B. serpentipileus* and *B. vulgaris*, by integrating NGS data with Sanger sequencing and morphological observation. Our results indicate that *B. serpentipileus* falls within the genus *Leccinum*, while *B. vulgaris* should be treated as a later synonym of *A. fusisporus*.

Regarding *B. serpentipileus*, Zang et al. (1999) [6] initially assigned this species to the genus *Boletellus* based on the morphological observation of distinctly longitudinally striate basidiospores. However, re-examination of the type specimen revealed basidiospores with a smooth, non-striate surface, measuring 16–19 × 4–6 µm, which differ from the original description. This morphological feature is inconsistent with *Boletellus*, but shows affinity with *Leccinum*, as the species also possesses a stipe entirely covered with brown squamules and smooth basidiospores. Accordingly, molecular phylogenetic analysis was conducted using DNA data obtained from this type specimen (KUN-HKAS31116) via NGS. The results demonstrate that the corresponding clade is unequivocally positioned outside the genus *Boletellus* and is nested within *Leccinum*. Based on combined morphological and molecular phylogenetic analyses, this study proposes the reclassification of this species into the genus *Leccinum*. Morphologically, *Leccinum serpentipileum* resembles *L. aurantiacum*. However, *Leccinum aurantiacum* has a pileus that is vivid red to red–brown and whitish context turning violaceous gray, gray, or blackish when bruised. Furthermore, it has smaller basidia (15–25.0 × 6.5–10.5 μm) and an intricate trichodermal pileipellis, with larger terminal elements often intracellularly granular-incrusted with red–brown pigment [47].

Early mycologists considered *Boletellus* to encompass species with basidiospores that were smooth (over 20 μm long), reticulate, verrucose, or longitudinally striate [48,49]. According to it, Bi et al. (1982) [7] initially assigned *B. vulgaris* to section *Allospori* of the genus *Boletellus* due to its fusiform basidiospores with verrucose ornamentation. However, the genus *Boletellus* was later delimited to encompass solely species exhibiting longitudinally striate ornamentation [50]. This refined definition necessitated a re-examination of the type specimen of *B. vulgaris*. Our re-observation confirmed that the basidiospore ornamentation is consistent with the description by Bi et al. (1982) [7]. However, the presence of pink tubes and reticulate stipe conflict with the modern delimitation of *Boletellus*. To clarify its phylogenetic position, NGS was employed in this study to obtain DNA data from this type specimen (GDGM4454). Molecular phylogenetic analysis demonstrated that *B. vulgaris* is nested within a clade that includes *A*. *fusisporus* in the genus *Austroboletus*. Based on morphological and molecular evidence, this study proposes to treat *B. vulgaris* as a synonym of *A. fusisporus.* Morphologically, they share several distinctive characteristics: small to minute basidiomata; a glutinous, fulvous pileus with an extended margin; pink tubes; a densely reticulate stipe surface; and basidiospores of similar size, ornamented with irregular, subcylindrical tubercles of varying sizes; cheilo- and pleurocystidia composed of two cells; and an intricate ixotrichoderm pileipellis.

This study, based on in-depth analyses of historical specimens of the genus *Boletellus*, validates the feasibility of next-generation sequencing technology in successfully getting the draft genomic data of aged specimens. This approach not only provides technical support for taxonomic study on historic herbarium collections, but also opens new avenues for exploring the potential of historical specimens in the studies on phylogeography and evolutionary history.

## Figures and Tables

**Table 1:** DNA sequences used in this study.

Species	Voucher	Locality	GenBank Accession No.					Reference
			ITS	nrLSU	*tef1-α*	*rpb1*	*rpb2*	
*Austroboletus* aff. *fusisporus*	KUN-HKAS52683	China	—	KF112484	KF112213	KF112571	KF112766	[16]
*A.* aff. *fusisporus*	KUN-HKAS53461	China	—	KF112486	KF112214	KF112572	KF112767	[16]
*A. albidus*	Feng1321	China	—	MT154755	—	—	—	[30]
*A. albidus*	ShiSF238	China	—	MT154756	—	—	—	[30]
*A. albovirescens*	KUN-HKAS59624	China	—	KF112485	KF112217	KF112570	KF112765	[16]
*A. albovirescens*	KUN-HKAS74743	China	—	KT990527	KT990730	—	KT990367	[4]
*A. amazonicus*	1839 AMV	Colombia	KF937307	KF714508	—	—	—	[31]
*A. amazonicus*	HUA 2032 AMV *	Colombia	NR_153523	NG_058569	—	—	—	[31]
*A. asper*	MEL2300520	Australia	KP242186	KP242253	—	—	—	—
*A. asper*	MEL2371703 *	Australia	KP242152	—	—	—	KP242055	—
*A. brunneisquamus*	FHMU5875	China	MZ855494	MW506828	—	—	—	[32]
*A. brunneisquamus*	FHMU5876 *	China	MZ855495	MW506829	MW512637	—	—	[32]
*A. fusisporus*	KUN-HKAS75207	China	JX889719	JX889720	JX889718	JX889721	—	[33]
*A. fusisporus*	122-549	Japan	AB509830	—	—	—	—	—
*A. fusisporus*	**KUN-HKAS1526** **86**	**China**	**PZ125049**	**PX930856**	**PZ137914**	—	—	**This study**
*A. fusisporus*	**KUN-HKAS1526** **93**	**China**	**PZ125048**	**PX930857**	**PZ137913**	—	—	**This study**
*A. lacunosus*	BRI:AQ0794285	Australia	KP242176	—	—	KP242103	—	—
*A. lacunosus*	BRI:AQ0795787	Australia	KP242161	KP242272	—	KP242050	KP242090	—
*A. mutabilis*	BRI:AQ0554076	Australia	KP242191	KP242229	—	—	—	—
*A. mutabilis*	BRI:AQ0554121	Australia	KP242192	KP242241	—	—	—	—
*A. niveus*	BRI:AQ0795772	Australia	KP242156	KP242276	—	—	—	—
*A. niveus*	REH9487	Australia	—	JX889668	JX889708	—	—	—
*A. novae-zelandiae*	PDD 101773	New Zealand	OP141471	OP141587	—	—	—	—
*A. novae-zelandiae*	PDD 83000	New Zealand	OP141462	OP141583	—	—	—	—
*A. olivaceobrunneus*	530828MF0042	China	—	MT154758	—	—	—	[30]
*A. olivaceobrunneus*	KUN-HKAS92428 *	China	—	NG_088132	MT110363	MT110400	MT110434	[30]
*A. olivaceoglutinosus*	KD 12-250 clone 1	India	KM597478	—	—	—	—	[34]
*A. olivaceoglutinosus*	KD 12-250 clone 2	India	KM597479	—	—	—	—	[34]
*A. rarus*	BRI:AQ0794045	Australia	KP242197	KP242236	—	KP242044	KP242086	—
*A. rarus*	BRI:AQ0807888	Australia	KP242200	—	—	—	—	—
*A. roseialbus*	Dodd	Australia	KY872653	KY872650	—	—	—	[35]
*A. roseialbus*	REH10024	Australia	KY872652	KY872651	—	—	—	[35]
*Austroboletus* sp.	MEL2305143	New Caledonia	KC552018	KC552060	KC552101	—	—	[36]
*Austroboletus* sp.	PL170519	New Caledonia	—	MZ827868	—	—	—	[36]
*A. subflavidus*	CFMR:BOTH-3463	USA	MT581521	MT580900	—	—	—	[37]
*A. subflavidus*	FLAS-F-60635	USA	MH016816		—	—	—	[37]
*A. viscidoviridis*	Perth 7588682	Australia	KP242219	KP242282	—	KP242074	KP242128	—
*A. viscidoviridis*	REH9993 *		KY872649	—	—	—	—	[35]
*Boletellus areolatus*	TNS-F-61560	Japan	AB989017	—	—	AB999747	AB999780	[38]
*B. areolatus*	TNS-F-61568 *	Japan	AB989021	—	—	AB999751	AB999784	[38]
*B. aurocontextus*	TNS-F-61562	Japan	AB989018	—	—	AB999748	AB999781	[38]
*B. aurocontextus*	TNS-F-61566 *	Japan	AB989020	—	—	AB999750	AB999783	[38]
*B. serpentipileus*	KUN-HKAS31116 *	**China**	—	**PZ000587**	**PZ137917**	**PZ137919**	**PZ137922**	**This study**
*B. verruculosus*	MHKMU H.Y.Huang 682 *	China	PP189882	PP179425	PP230535	PP195249	PP19526	[39]
*B. verruculosus*	MHKMU H.Y.Huang 699	China	PP189881	PP179426	PP230536	PP195250	PP195263	[39]
*B. vulgaris*	**GDGM4454**	**China**	**PX986931**	**PZ000589**	—	—	**PZ137924**	**This study**
*B. wenshanensis*	KUN-HKAS122937	China	—	ON006511	ON007375	—	—	[40]
*B. wenshanensis*	KUN-HKAS122938 *	China	—	ON006512	ON007378	ON007376	ON007375	[40]
*Lanmaoa asiatica*	KUN-HKAS63516	China	—	KT990584	KT990780	KT990935	KT990419	[4]
*Lanmaoa asiatica*	KUN-HKAS63603	China	—	KM605143	KM605153	KM605165	KM605176	[4]
*Leccinum. aurantiacum*	L:0342207	France	—	MK601759	MK721113	—	MK766318	[41]
*L. aurantiacum*	**KUN-HKAS1542141**	**France**	—	**PX987021**	**PZ137915**	—	**PZ102461**	**This study**
*L. aurantiacum*	**KUN-HKAS1542142**	**France**	**PX990527**	**PZ000588**	**PZ137918**	**PZ137920**	**PZ137923**	**This study**
*L.* cf. *duriusculum*	CFMR:DLC2002-252	USA	—	MK601760	—	—	MK766336	[41]
*L.* cf. *duriusculum*	MICH:KUO-09120708	USA	—	MK601761	—	—	MK766320	[41]
*L. duriusculum*	Yang5971	Austria	—	MZ675541	MZ707785	—	MZ707779	[42]
*L. holopus*	CFMR:BOS-569	USA	—	MK601762	MK721116	—	MK766321	[41]
*L. holopus*	MICH:KUO-09150707	USA	—	MK601763	MK721117	—	MK766322	[41]
*L. monticola*	KUN-HKAS76669	China	—	KF112443	KF112249	KF112592	KF112723	[16]
*L. monticola*	NY:00815448	Costa Rica	—	MK601767	MK721121	—	MK766326	[41]
*L. monticola*	NY:760388	Costa Rica	—	MK601766	MK721120	—	MK766325	[41]
*L. pseudoborneense*	Zhao476	China	—	MZ536631	MZ543306	—	MZ543308	[42]
*L. pseudoborneense*	Zhao773	China	—	MZ536632	MZ543307	—	MZ543309	[42]
*L. scabrum*	KUN-HKAS53604	China	—	KT990586	—	KT990938	KT990422	[4]
*L. scabrum*	KUN-HKAS56371	China	—	KT990587	KT990782	—	KT990423	[4]
*L. scabrum*	KUN-HKAS57266	China	—	KF112442	KF112248	—	KF112722	[16]
*L. serpentipileum*	LJW270	China	MZ485405	MZ675540	MZ707784	—	MZ707778	[42]
*L. serpentipileum*	**KUN-HKAS123627**	**China**	**PZ127545**	**PX930858**	**PZ137916**	**—**	**PZ137921**	**This study**
*Leccinum* sp.	BJTC FM2191	China	—	OR655198	OR659998	—	OR659950	[43]
*Leccinum* sp.	BJTC FM2419	China	—	OR655199	OR659999	—	OR659951	[43]
*L. versipelle*	CFMR:DLC2002-122	USA	—	MK601778	—	—	MK766336	[41]
*L. versipelle*	FB27	China	—	MZ675546	MZ707790	—	MZ707782	[42]
*L. versipelle*	KPM-NC-0017833	United Kingdom	—	JN378514	JN378454	—	—	[44]
*L. versipelle*	LJW418	China	—	MZ675545	MZ707789	—	MZ707781	[42]
*L. vulpinum*	KPM-NC-0017834	United Kingdom	—	JN378516	JN378456	—	—	[44]

Type specimens are marked with an asterisk (*). The bold formatting indicates the newly generated data in this study.

## Data Availability

The original contributions presented in this study are included in the article; further inquiries can be directed to the corresponding authors.
